# Computational Biomechanical Analysis of Postoperative Calcaneal Fractures with Different Placement of the Sustentaculum Screw

**DOI:** 10.1111/os.12541

**Published:** 2020-02-13

**Authors:** Min‐fei Qiang, Ritesh Kumar Singh, Yan‐xi Chen, Kun Zhang, Xiao‐yang Jia, Song Chen, Shu‐guang Wang, Xiong Wang, Zhao‐man Shi

**Affiliations:** ^1^ Department of Orthopaedic Surgery Zhongshan Hospital, Fudan University Shanghai China; ^2^ Department of Orthopaedic Trauma Shanghai East Hospital, Tongji University School of Medicine Shanghai China

**Keywords:** Calcaneus, Finite element analysis, Fracture fixation, Internal, Intra‐articular fractures

## Abstract

**Objective:**

To evaluate the computational biomechanical analysis of intra‐articular calcaneal fractures with different fixation status of the sustentaculum plate screw, when the finite element modeling of calcaneal fractures were fixed by the lateral locking plate.

**Methods:**

The normal right foot of a male (age: 36 years; height: 174 cm; body weight: 65 kg) was scanned by the CT scanner. As the computational biomechanical study, the three‐dimensional finite element model of the simplified Sanders type‐II calcaneal fracture was built. Fixation with the lateral calcaneal locking plate and screws was simulated using a finite element software package according to clinical operation. According to the different placement of the sustentaculum plate screw, the models were categorized as the accurate fixation group, marginal fixation group, and non‐fixation group. The loading of 650 N with the vertical axial compression was applied to simulate the standing phase with single foot. The Von Mises stress distribution, maximal displacement, and contact area of the subtalar joint were analyzed among three groups.

**Results:**

The pressure distribution of the subtalar joint facet was inhomogeneous. The stress concentration of the calcaneus was located at the medial zone of the posterior subtalar joint facet. The peak Von Mises stress distribution in three groups was similar at the subtalar joint facet of 4.9 MPa, 5.1 MPa, and 5.4 MPa. In the accurate fixation group, the contact area on the posterior articular facet was 277.1 mm^2^; the maximal displacement was 0.18 mm. The contact area of the marginal fixation group was 265.3 mm^2^ on the posterior facet, where the maximal displacement was 0.23 mm. In the non‐fixation group, the contact area was 253.8 mm^2^; the maximal displacement was 0.25 mm. There was a slight change in the contact area of the subtalar joint and no prominent displacement of the calcaneus could be detected among the three groups.

**Conclusions:**

The biomechanical results, including the peak stress distribution, contact area, and maximal displacement of subtalar joint, were similar whether the screw is placed exactly within the sustentaculum tali or not, when the calcaneal fractures were fixed by the lateral locking plate. The sustentaculum plate screw had less effect on the biomechanical performance of the calcaneus.

## Introduction

As the common tarsal bone fracture, calcaneal fractures account for approximately 2% of all fractures, and three‐fourths of calcaneal fractures are intra‐articular^1^. Surgical treatments, including open reduction and internal fixation (ORIF) and a variety of minimally invasive osteosynthesis, are generally recommended for the displaced intra‐articular calcaneal fractures^2^, ^3^. As the gold standard, ORIF is considered as the ideal method for achieving anatomic reduction^2^, ^3^, ^4^. The surgical treatment of the intra‐articular calcaneal fractures could be performed through various approaches. Currently, the lateral calcaneal plate fixation through the lateral approach is commonly used^4^, ^5^. After the fracture, fragments were anatomically reduced through the lateral approach, the lateral plate was attached to the calcaneal lateral wall, and then the screws inserted through the plate holes were placed into the calcaneus, including the anterior process, the subthalamic area, and the tuberosity^6^, ^7^.

As a special structure of the calcaneus, the sustentaculum tali plays an important role in supporting transmission stress in the weight bearing of the foot. Therefore, in the subthalamic area of the lateral calcaneal plate, one sustentaculum screw was often inserted through the plate hole and beneath the posterior facet of the subtalar joint into the sustentaculum tali so as to acquire stable fixation of medial segment. However, correct insertion of this sustentaculum screw was sometimes technically difficult^8^. During the operation, the sustentaculum screw inserted through the lateral plate would possibly increase the exposure time of intraoperative fluoroscopy, prolong operative time, and increase the risk of injuring the medial neurovascular bundle. This is because the posterior tibial neurovascular bundle near the sustentaculum tali runs adjacent to the medial border of the calcaneus. The direct visualization of the sustentacular screw holding the reduction is unavailable through the extended lateral approach. The screw or Kirschner wires misdirected to the sustentaculum tali, or too long a screw, may damage the neurovascular bundle during the reduction of the medial fragments, to which surgeons should pay attention^9^.

In the recent years, with the development and improvement of calcaneal locking plates, it makes the clinician confused whether the sustentacular screw is still necessary or not during the operation. Theoretically, these locking plates provide good coronal plane stability of the calcaneus, and they may provide stable support of the medial segment as well. So far, various kinds of biomechanical studies have been done on calcaneal fractures based on cadaver specimens or models, while the internal stress transmission mechanism of the bone stress can be difficult to reveal^10^, ^11^. Compared with the limitation of source shortage of specimens, and the inability to perform the research with different load conditions on cadaver specimens or physical models, the three‐dimensional (3‐D) finite element analysis (FEA) method has become one of the most effective approaches for biomechanical studies due to the rapid development of computer technology. As a reliable and effective computational biomechanical tool, FEA could be used to calculate the stress distribution, the contact area, and the displacement of the specific model^12^, ^13^, ^14^. Ni *et al*.^14^ constructed a simplified finite element model of the calcaneus to assess the absorbable screw fixation for intra‐articular calcaneal fractures, including the Von Mises stress of the screws and the displacement of the calcaneus and screws. The previous computational biomechanical study introduced a 3‐D finite element model of inferior tibiofibular syndesmosis that was made up of ankle, hindfoot, and ligaments to analyze the biomechanical effect of the syndesmotic screw^12^. And the modeling method for finite element models has been modified for saving more time and realizing personalized modeling for clinical application.

The observational study based on 3‐D CT has indicated that the ratio of sustentaculum screw acquiring accurate fixation was not high when the calcaneal fractures were treated by ORIF *via* the lateral approach; however, the clinical outcomes were similar whether the screw was accurately placed within the sustentaculum or not^15^. Therefore, the aims of this study are: (i) to present the 3‐D finite element modeling of the postoperative calcaneal fracture; (ii) to simulate specific conditions in terms of different placement of the sustentaculum screw; and (iii) to perform the computational biomechanical analysis. We hypothesize that the different fixation status of the sustentaculum screw has less effect on the biomechanical results of the subtalar joint when the intra‐articular calcaneal fractures are treated by the lateral locking plate.

## Materials and Methods

### *Participant and CT Scanning*

The biomechanical evaluation was conducted on a 3‐D model collected from a healthy volunteer. The normal right foot of a male (age: 36 years; height: 174 cm; body weight: 65 kg) without deformity, osteoarthritis, or tumor, was scanned by the CT scanner (Philips Brilliance 64 CT Scan, Philips Medical Systems, Netherland). The study was approved by the institutional review committee of the hospital and performed with the written, informed consents from the volunteer. The experiment was carried out in accordance with guidelines of the Declaration of Helsinki. Imaging parameters of CT scan included: section thickness, 1.0 mm; pitch, 1.375; tube voltage, 120 kV; matrix, 512 × 512. All CT data of the volunteer was collected from the medical image database in the hospital, which were saved in the DICOM 3.0 format (.dcm).

### *3‐D Reconstruction of Calcaneus*

**Figure 1:**
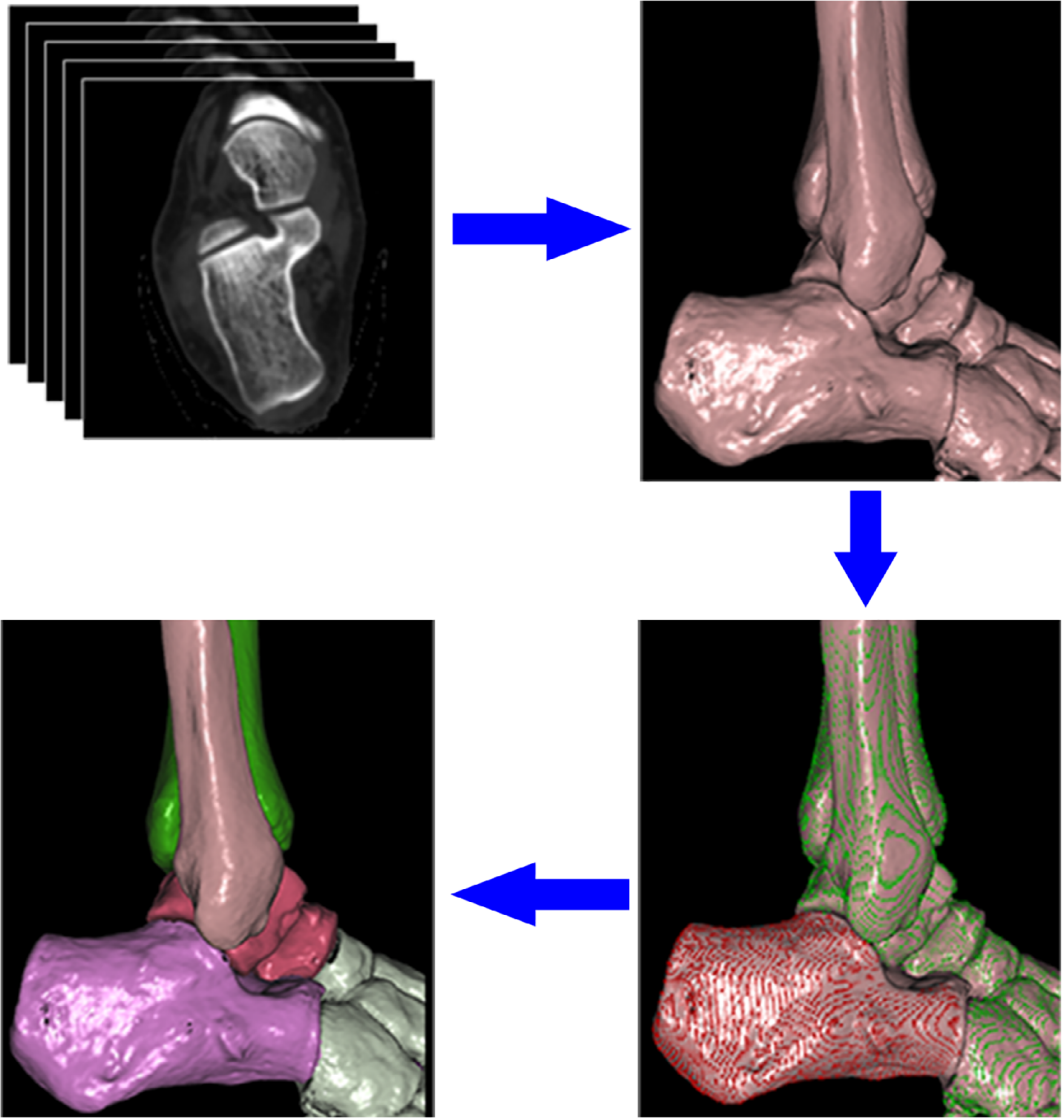
The flow diagram of rapid 3‐D segmentation of the hindfoot. The CT data (DICOM 3.0 format) was imported into software, SuperImage. The calcaneus and other bony structures were divided quickly by 3‐D interactive and automatic segmentation techniques, which saved a lot of time in the modeling process.

Thin‐slice CT transverse images of the calcaneus were firstly uploaded to the picture archiving and communication systems (PACS). And then these CT data were entered into the digital orthopedic clinical research platform (SuperImage orthopedics edition 1.1, Cybermed Ltd., Shanghai, China), in which the bone contour was obtained^12^, ^16^. The 3‐D modeling of calcaneus was reconstructed by shaded surface display (SSD) with a reconstruction interval of 0.625 mm. All component bones of the hindfoot were distinguished by 3‐D interactive and automatic segmentation technique. The model was exported with a form of point cloud (Fig. 1).

The solid model of the calcaneus was constructed in software Solidworks 2014 (SolidWorks Corporation, MA, USA). After denoising, smoothing, and surface fitting, the geometric model with non‐uniform rational basis splines surface for bony structures of the calcaneus was created. The anatomical calcaneal plate, locking screws and cortex screws with the diameter of 3.5 mm were created by the reverse engineering function. These geometric models, including bones, the plate, and screws, were exported as the format of IGES.

### *Preprocessing of Finite Element Modeling*

**Figure 2:**
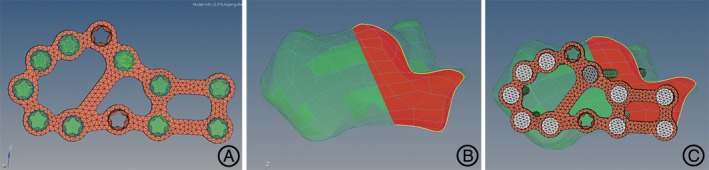
Reconstructed model of the intra‐articular calcaneal fracture. (A) The lateral calcaneal locking plate and screws were selected. (B) The Sanders type‐II calcaneal fracture was simulated. (C) The fracture model was fixed after the virtual surgery.

The geometric models for preprocessing FEA were assembled with Hypermesh 12.0 (Altair Engineering, Michigan, USA). Bon structures of the hindfoot were meshed with tetrahedral elements. According to the previous study, grid density was set to “good,” grid parameters to “normal grid,” overall size to 2.8 mm, tolerance to 0.1 mm, and node of Jacobi to four^12^. In consideration of the minimal effect on the predicted contact pressure, the articular cartilage was created by offsetting function and the thickness of the mesh of the joint surface was set as 1.8 mm^17^, ^18^. According to the previously published literature^12^, ^18^, ^19^, Young's modulus and Poisson's ratio were set and listed in Table 1. The bone structures of the hindfoot were simplified as homogeneous and isotropic linear elastic. The Sanders type‐II calcaneal fracture was simulated and fixed by the lateral calcaneal locking plate, where the cortical screw was toward the sustentaculum tali and the other screws were locked (Fig. 2).

**Table 1:** The mechanical properties of the finite element model

	Cortical bone	Cancellous bone	Internal fixation devices
Young's modulus	7300 MPa	1450 MPa	200000 MPa
Poisson's ratio	0.30	0.20	0.33

**Figure 3:**
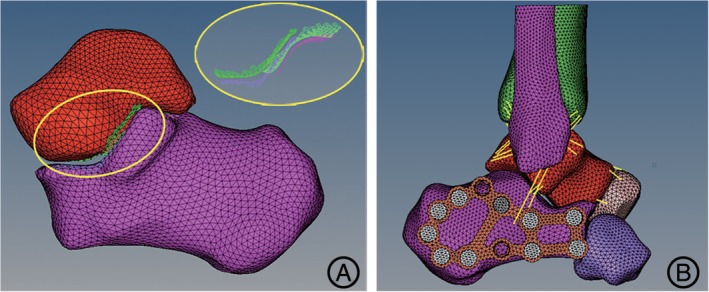
The contact of the articular surface and the rebuilding of ligaments. (A) The contact pairs of subtalar joint were stimulated by the automated surface‐to‐surface contact algorithm. (B) The 3‐D finite element model containing the ligaments was fixed with the lateral locking plate.

The contact of the articular surfaces was simulated by the contact pairs which were set as the type of hard contact without penetration^20^. The automated surface‐to‐surface contact algorithm based on Lagrange multipliers to simulate the contact between the joint surfaces was conducted in the software ABAQUS 6.14 (Dassault Systèmes, RI, USA). The contact pairs of subtalar joints were built up (Fig. 3A). With regard to the rebuilding of ligaments, such as calcaneofibular ligament, talocalcaneal ligament, deltoid ligament, and talofibular ligaments, the 1D unit truss (T3D2) was applied to simulate the tensile ligament fibers with no compression in Hypermesh software 12.0 (Altair Engineering, Michigan, USA) (Fig. 3B).

### *Boundary and Loading Conditions*

To set up the boundary and loading conditions, the posterior end of the calcaneal tuberosity was constrained. The talocalcaneonavicular and calcaneocuboid joints were restrained in the anterior–posterior direction. The loading of 650 N with the vertical axial compression through the talus was applied to simulate the standing phase with single foot. The fractured segments for contact was assigned with a coefficient of friction of 0.2; and the contact between the calcaneus and the plate was set as frictionless^21^. According to the different placement of the sustentaculum plate screw, the models were divided into three groups: the accurate fixation group, the marginal fixation group, and the non‐fixation group, each of which was described in the previous study^15^.

### *Validation of Calcaneus Finite Element Model*

The construct stiffness of the intact calcaneus model and the fracture model fixed by locking plate fixation was predicted to validate the finite element model. The construct stiffness was compared with the cadaveric calcaneus experiment under the similar experimental conditions of 700 N vertical loads reported by Ni *et al* 22.

### *Indexes of Finite Element Calculation*

The distribution characteristics of the Von Mises stress on the subtalar joint facet was displayed and the peak stress was calculated by the FEA software ABAQUS 6.14 (Dassault Systèmes, RI, USA). The contact area of the articular surface and the maximal displacement at the subtalar joint along the loading direction were also recorded in three groups.

## Results

### *Model Validation*

The finite element model of the calcaneus simulating the one‐leg standing phase was validated as follows. The finite element model predicted the highest vertical construct stiffness of the intact calcaneus (618 N/mm under 650 N vertical loads) was nearly coincident with the construct stiffness of the cadaveric experiment (634 N/mm under 700 N vertical loads) from the published study^22^. The FEA showed the locking plate fixation for the fracture model provided the highest construct stiffness value of 473 N/mm, which was also similar with the stiffness value of 440 N/mm from the cadaveric experiment^22^.

### *Peak Stress Distribution*

**Figure 4:**
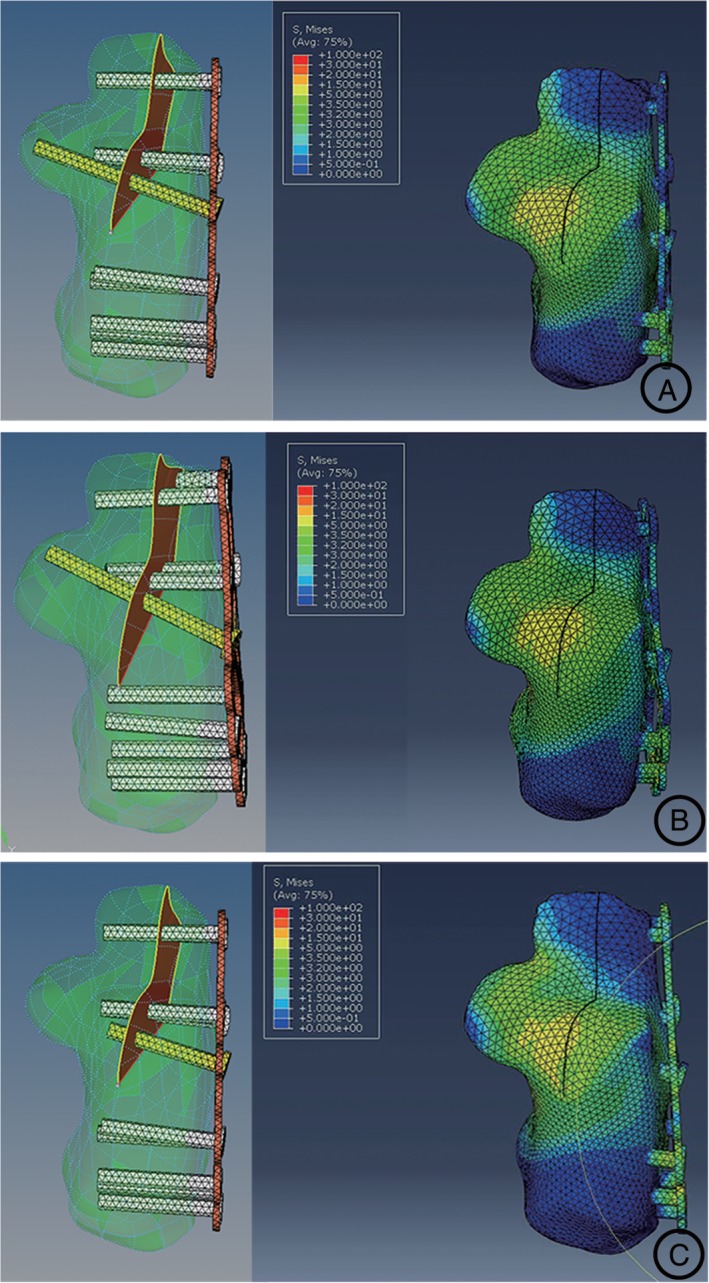
Results of FEA of the models. According to the placement of the sustentaculum plate screw, the stress distribution was measured in three groups: (A) the accurate fixation group; (B) marginal fixation group; and (C) non‐fixation group. And in every group, the stress concentration of the calcaneus was similarly located at the medial zone of the posterior facet.

The 3‐D finite element modeling of the calcaneal fracture fixed with the lateral anatomical locking plate was established. After the vertical axial loading of 650 N and the boundary conditions were set, the FEA of the subtalar joint was realized. Among the three groups, the peak Von Mises stress distribution was similar at the subtalar joint facet of 4.9 MPa, 5.1 MPa, and 5.4 MPa (Table 2). The general trend for the pressure distribution of the subtalar joint facet was inhomogeneous. With respect to the location of the pressure centroids, the pressure mainly concentrated on the medial zone of the posterior subtalar joint facet in each group (Fig. 4).

**Table 2:** The peak stress, contact area and displacement of subtalar joint

	Accurate fixation group	Marginal fixation group	Non‐fixation group
Peak stress (MPa)	4.9	5.1	5.4
Contact area (mm^2^)	277.1	265.3	253.8
Displacement (mm)	0.18	0.23	0.25

### *Contact Area and Maximal Displacement*

The contact area and the maximal displacement of the subtalar joint in every group were summarized in Table 2. In the accurate fixation group, the contact area on the posterior articular facet was 277.1 mm^2^; the maximal displacement of the calcaneus was 0.18 mm. The contact area of the marginal fixation group was also on the posterior facet of 265.3 mm^2^, where the maximal displacements was 0.23 mm. In the non‐fixation group, the contact area on the posterior facet was 253.8 mm^2^; the maximal displacement was similarly 0.25 mm. No prominent displacement of the subtalar joint could be detected among the three groups.

## Discussion

The sustentaculum tali is attached to the medial malleolus by the deltoid ligament and, together with the talus, it forms the lateral boundary of the tarsal tunnel. Due to the stable surrounding structures, the sustentaculum is often separated from the calcaneus body in the calcaneal fracture. The flexor hallucis longus and posterior tibial tendon run adjacent to the sustentaculum tali. The flexor hallucis longus tendon is below the inferior groove of the sustentaculum tali, when it passes to the sole of the foot^23^. During the surgical treatment for calcaneal fractures through the lateral approach, the middle plate screw under the posterior facet is inserted toward the sustentaculum tali in order to acquire stable fixation of the medial fragments after the displaced posterior facet are reduced^7^. In the previous investigation of the screw placement for calcaneal fractures, the screw accurately inserted within the sustentaculum tali was not in the majority. However, the rate of anatomic reduction and the clinical outcomes were similar whether the screw was exactly placed within the sustentaculum or not^15^.

### *FEA Studies on Calcaneal Fracture*

Yu *et al*.^24^ compared the biomechanical performance of the conventional and anatomical calcaneal plates fixation for calcaneal fractures. The calcaneal plate with an additional sustentaculum tali screw fixation outside the plate or without the additional screw fixation was simulated, and the stress and displacement distributions of two kinds of fixation devices were analyzed^25^. However, the computational biomechanical analysis of the calcaneal fractures with different sustentaculum screws through the lateral locking plate holes has not been reported. In the present study, a 3‐D finite element model of calcaneus was established to analyze the stability of intra‐articular calcaneal fractures treated by the lateral calcaneal plate with different fixation status of the sustentaculum screw. The stress and displacement distributions of the calcaneus fragments during the standing phase of single foot were obtained. The FEA results revealed that the stress concentration of the subtalar joint was located at the medial zone of the posterior facet. The posterior facet of the subtalar joint was thus the main support structures for the vertical axial loading.

### *Stress of Subtalar Joint and Maximal Displacement*

When the data of the stress of subtalar joint was obtained, the stress concentration appeared at the medial part of the posterior facet, which could be the inducement of fragment re‐displacement or internal fixation loosening after the superimposed loading of the hindfoot. The peak stress was similar among the three groups, as well as the maximal displacement of the calcaneus. Furthermore, the maximal displacement ranged from 0.18 to 0.25 mm, which was not notable. The results indicated that the calcaneal locking plates were stable for calcaneal fracture fixation. It might be related to the fact that the calcaneal locking plates provide enough coronal plane stability to support the medial fragment^26^. Patients with calcaneal fractures fixed by the lateral plate are usually encouraged to start the ankle and subtalar joint mobilization exercises soon after surgery. However, full weight bearing exercises, such as running, should be avoided for a short period after surgery, which can also reduce the risk of the fixation loosening, because the loading on hindfoot would be multiplied compared with the standing phase.

### *Contact Area of the Posterior Facet*

The biomechanical study demonstrated that the contact area of the posterior facet of the subtalar joint was concerned with the reduction quality of the fracture^27^. The displacement or step‐offs of more than 1–2 mm would cause a pressure redistribution within the posterior facet in the simulated calcaneal fracture patterns^27^, ^28^. The quality of reduction of the subtalar joint facet after operative management was regarded as the important fact for the outcome of calcaneal fractures. Buckley *et al*.^4^ reported that the patients with anatomic reduction of the posterior facet or incongruity of less than 2 mm had good functional outcomes. The larger the displacement of the posterior facet, the smaller the contact area would be. In this study, there was no prominent displacement of the subtalar joint among the three groups, and the contact area was similar whether the sustentaculum plate screw was exactly placed or not.

### *Limitations of the Study*

There were also some limitations of this study. Firstly, on account of the difficulty in determining the typical values for mechanical characterization of human tissues, the muscle and soft tissues were neglected during the modeling process. Secondly, only the simplified Sanders type‐II calcaneal fracture was simulated in the FEA. Further computational biomechanical research about other types of calcaneal fractures would be carried out in the future. Finally, due to nonlinear problems in the modeling, the convergence of results was hard to control. Therefore, the bone structures were simplified as homogeneous and isotropic linear elastic in the modeling process.

### *Conclusions*

In this study, we constructed a 3‐D finite element model of the lateral locking plate for intra‐articular calcaneal fractures with different fixation status of the sustentaculum plate screw. However, correct insertion of the sustentaculum screw is technically difficult in clinical practice. Based on the results of FEA, the sustentaculum plate screw had less effect on the biomechanical performance of the calcaneus. The biomechanical results, including the peak stress distribution, contact area and maximal displacement of subtalar joint, were similar whether the screw is exactly placed within the sustentaculum tali or not, when the calcaneal fractures were fixed by the lateral locking plate.
